# High-Resolution U-Net: Preserving Image Details for Cultivated Land Extraction

**DOI:** 10.3390/s20154064

**Published:** 2020-07-22

**Authors:** Wenna Xu, Xinping Deng, Shanxin Guo, Jinsong Chen, Luyi Sun, Xiaorou Zheng, Yingfei Xiong, Yuan Shen, Xiaoqin Wang

**Affiliations:** 1 Center for Geo-Spatial Information, Shenzhen Institutes of Advanced Technology, Chinese Academy of Sciences, Shenzhen 518055, China; wn.xu@siat.ac.cn (W.X.); xp.deng1@siat.ac.cn (X.D.); sx.guo@siat.ac.cn (S.G.); ly.sun@siat.ac.cn (L.S.); xiaorou.zheng@siat.ac.cn (X.Z.); yf.xiong@siat.ac.cn (Y.X.); yuan.shen@siat.ac.cn (Y.S.); 2 University of Chinese Academy of Sciences, Beijing 101407, China; 3 Shenzhen Engineering Laboratory of Ocean Environmental Big Data Analysis and Application, Shenzhen 518055, China; 4 Key Laboratory of Spatial Data Mining & Information Sharing of Ministry of Education, National & Local Joint Engineering Research Center of Satellite Geospatial Information Technology, Fuzhou University, Fuzhou 350000, China; wangxq@fzu.edu.cn

**Keywords:** full convolutional network, U-Net, cultivated land extraction, deep learning, remote sensing

## Abstract

Accurate and efficient extraction of cultivated land data is of great significance for agricultural resource monitoring and national food security. Deep-learning-based classification of remote-sensing images overcomes the two difficulties of traditional learning methods (e.g., support vector machine (SVM), K-nearest neighbors (KNN), and random forest (RF)) when extracting the cultivated land: (1) the limited performance when extracting the same land-cover type with the high intra-class spectral variation, such as cultivated land with both vegetation and non-vegetation cover, and (2) the limited generalization ability for handling a large dataset to apply the model to different locations. However, the “pooling” process in most deep convolutional networks, which attempts to enlarge the sensing field of the kernel by involving the upscale process, leads to significant detail loss in the output, including the edges, gradients, and image texture details. To solve this problem, in this study we proposed a new end-to-end extraction algorithm, a high-resolution U-Net (HRU-Net), to preserve the image details by improving the skip connection structure and the loss function of the original U-Net. The proposed HRU-Net was tested in Xinjiang Province, China to extract the cultivated land from Landsat Thematic Mapper (TM) images. The result showed that the HRU-Net achieved better performance (Acc: 92.81%; kappa: 0.81; F1-score: 0.90) than the U-Net++ (Acc: 91.74%; kappa: 0.79; F1-score: 0.89), the original U-Net (Acc: 89.83%; kappa: 0.74; F1-score: 0.86), and the Random Forest model (Acc: 76.13%; kappa: 0.48; F1-score: 0.69). The robustness of the proposed model for the intra-class spectral variation and the accuracy of the edge details were also compared, and this showed that the HRU-Net obtained more accurate edge details and had less influence from the intra-class spectral variation. The model proposed in this study can be further applied to other land cover types that have more spectral diversity and require more details of extraction.

## 1. Introduction

Accurate area and change of cultivated land is one of the fundamental types of data for precision agriculture, food security analysis, yields forecasting, and land-use/land-cover research [1]. In the arid and semi-arid regions, this information is particularly important as it is related to the regional water balance and the local ecosystem health [2]. Currently, the increasing free remote sensing data (such as U.S. Geological Survey (USGS) Landsat and European Space Agency (ESA) Sentinel) provides sufficient data sources and the opportunity to extract and monitor the dynamic change of the cultivated land [3,4,5].

However, the cultivated land, as a man-made concept, usually shows different spectral characteristics due to the varying types of crops, different irrigation methods, and different soil types, as well as fallow land plots. As a result, for classification, the intra-class variation increases and the inter-class separability decreases [6,7]. The frequently used traditional pixel-based classifiers, such as support vector machine (SVM), K-nearest neighbors (KNN), and random forest (RF) [8,9], and the object-based farmland extraction models, such as the stratified object-based farmland extraction [6], the superpixels and supervised machine-learning model [10], and the time-series-based methods [11], usually require the prior knowledge to model the high intra-class variation of the spatial or spectral features. Due to this, the features learned by these methods are often limited to the specific datasets, time, and locations, which is known as limited model generalization ability. The re-training process is usually required when applying these models to different datasets, time, and locations.

With the rapid development of deep learning [12], convolutional neural networks (CNNs) have gained state-of-the-art performance in land cover classification, which overcomes the abovementioned difficulties [13]. Possible reasons for its success include: (1) the capacity of learning from a large dataset; (2) the tolerance for larger intra-class variation of the object features; and (3) the high generalization ability. Benefitting from the large training dataset, the feature variation of the target object across different locations or time can be modeled. CNNs have shown great advantage in urban land use and land cover mapping [14,15,16,17,18], scene classification [19,20,21], and object extraction [22,23,24,25]. Among the popular CNNs, the U-Net is reported to achieve state-of-the-art performance on several benchmark datasets even with limited training data [26,27]. It was widely used in many fields as a result.

However, the “pooling” process in most deep convolutional networks, which (1) provides the invariance (translation, rotation, and scale-invariance) capacity for the model to capture the major feature of the target; (2) reduces the number of parameters for multi-scale training; and (3) increases the receptive field by involving the down-sampling process (converting images from the high to low spatial resolution) with certain calculation (maximum, average, etc.), leads to significant detail loss from the image, including edges, gradients, and image texture details [28,29]. This problem could decrease the accuracy of extraction of land cover type even more when dealing with remote sensing images considering the high intra-class spectral variation [30]. Ideas to solve this problem can currently be organized in two categories: (1) learning and recovering high-resolution details from low-resolution feature maps or (2) maintaining high-resolution details throughout the network [31].

In the first category, the detailed texture is recovered from the low-resolution feature maps after the pooling process by applying certain up-sampling methods (such as bilinear interpolation or deconvolution) [32,33,34] of the representative model in this category, such as the fully convolutional network (FCN) [35], In SegNet [36], DeconvNet [37], RefineNet [38] et al.

For remotely sensed images, this idea was widely used. For instance, the FCN-based network achieved an overall accuracy of 89.1% on the International Society for Photogrammetry and Remote Sensing (ISPRS) Vaihingen Dataset without a down-sampling layer to obviate deconvolution in the latter part of the structure [39]. Marmanis, et al. (2016) designed a segmentation network at the pixel-level that synthesized the FCN and deconvolution layers and refined the results using fully connected conditional random fields (CRF) [40]. ASPP-Unet and ResASPP-Unet recovered the spatial texture by adding the Atrous Spatial Pyramid Pooling (ASPP) technique in network to increase the effective field-of-view in convolution and capture the features in multiple scales [41]. MultiResoLCC provides a two-branch CNN architecture to improve the image details by jointly using panchromatic (PAN) and multi spectral (MS) imagery [42]. For hyperspectral image classification tasks, the CNN structure can also improve the accuracy by extracting the hierarchical features [43] and creating the low-dimensional feature space to increase the separability [44].

This type of method recovers the high-resolution details by learning from low-resolution feature maps. Although various skip connection methods have been used to optimize the obtained high-resolution details, the effect is limited since the lost details are usually recovered only from low-spatial resolution features. This often causes the recovering procedural to be ill-posed as the number of pixels of the output is always bigger than that of the input.

In the second category, high-resolution details are first extracted and maintained through the whole process, typically by a network that is formed by connecting multi-level convolutions with repeated information exchange across parallel convolutions. Under this idea, the skip connection is usually redesigned between the pooling nodes and the up-sampling nodes. For instance, (1) adding more skip connections to link different convolution nodes at the same scale, and (2) adding more skip connections to link the convolution nodes at the different scales. Representative models include convolutional neural fabrics [45], interlinked CNNs [46], and high-resolution networks (HRNet) [47]. This kind of method avoids the ill-posed problem; however, the time consumed in the training process can dramatically increase. More free parameters in the model require more data to train.

In this paper, we propose a new end-to-end cultivated land extraction algorithm, high-resolution U-Net (HRU-Net), to extract cultivated land from Landsat TM images. The new network is based on the U-Net structure, in which the skip connections are redesigned following the ideas of the second category mentioned above to obtain more details. Inspired by HRNet, the loss function of the original U-Net is also improved to take into account features extracted at both shallow and deep levels. The proposed HRU-Net was tested in Xinjiang Province, China for the cultivated land extraction based on three years’ worth of Landsat TM images, and was compared with the original U-Net, U-Net++, and the RF method. The major contributions of this study can be summarized as: (1) we redesigned the skip connection structure of the U-Net to keep the high-resolution details for remote sensing image classification; (2) we modified the original U-Net loss function to achieve a higher extraction accuracy for the target with a high intra-class variation; (3) we proposed a new end-to-end cultivated land extraction algorithm, the high-resolution U-Net (HRU-Net), which demonstrated good performance in extracting the target with high edge details and high intra-class spectral variation.

## 2. Related Work

### 2.1. Learning and Recovering High-Resolution Details from Low-Resolution Feature Maps

The representative model in this category is the fully convolutional network (FCN) [35]. In each stage of an FCN, an up-sampling subnetwork, like a decoder, was used as the up-sampling procedure, which attempts to recover the fine-spatial resolution details from the coarse-spatial resolution feature maps [33,34,48]. In SegNet [36], the up-sampling strategy is a mirrored symmetric version from the pooling subnetwork by grabbing the indices directly for the pooling subnetwork. The up-sampling strategy can be combined with the deconvolution process, such as in DeconvNet [37], where the locations and values of the highest gradience are kept by the up-sampling strategy, and the sparseness of the up-sampling output is repaired by the deconvolution layers. In RefineNet [38], instead of using only one feature map from one pooling layer, the long-range residual connections were used to combine all information along with all pooling layers to refine the high-resolution details. Other asymmetric structures, such as the light up-sampling process [49], light pooling, heavy up-sample processes [50], and re-combinator networks [51], were all reported have good performance for object detection.

### 2.2. Maintaining High-Resolution Details throughout the Network

Representative models include convolutional neural fabrics [45], interlinked CNNs [46], and high-resolution networks (HRNet) [47]. In an HRNet, a high-resolution subnetwork was first established as the first stage, then the high-to-low resolution subnetworks were added consecutively to form more low-level stages. This structure maintains the high-resolution details through the whole process and has achieved state-of-the-art performance in the field of human pose estimation [47]. Fu et al. (2019) and Wu et al. (2018) also improved skip connections by stacking multiple DeconvNets/UNets/Hourglasses with dense connections [52,53].

## 3. Study Area and Datasets

**Figure 1:**
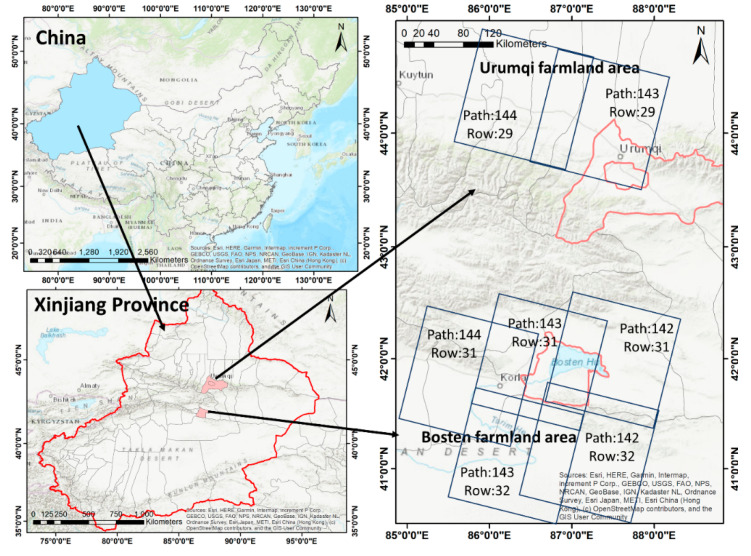
The study area.

In this paper, the intra-class spectral variation of cultivated land can be reflected in three perspectives: (1) intra-class spectral variation over different time, (2) intra-class spectral variation over different geo-locations, (3) intra-class spectral variation over different crop types. These three variation factors can be represented with multiple times (both winter and summer) and different locations within a large area. The study area is located in the Urumqi and Bosten farmlands in Xinjiang, China (Figure 1), which mainly grow cash crops, such as cotton and pears. The crops are planted in large areas with high yield and require a huge amount of water supply every year. Extracting cultivated land of these two regions is of great significance to the agricultural and water resource monitoring to ensure the national food security of Xinjiang and China.

Landsat5 thematic mapper (TM) top of atmosphere (TOA) reflectance (the USGS Earth Explorer: https://earthexplorer.usgs.gov/) from 2009 to 2011 was collected as the dataset in this study. The TM sensor has seven spectral bands (Table 1), but we only selected six bands with a resolution of 30 m: B1 (blue), B2 (green), B3 (red), B4 (near-infrared, NIR), B5 (short-wave-infrared, SWIR 1), and B7 (short-wave-infrared, SWIR 2). The thermal band was not used in this study as it could vary during the different observation dates, which was caused by the different local environmental factors, such as the radiative energy the land received or the wind speed. Only cloud free images were chosen in this study. The image details are shown in Table 2.

We used the historical landcover map in the 2010 version from the local government to extract the ground truth manually based on the Landsat 5 image at 30 m scale. The changes in the land cover types were considered to be consistent from 2009 to 2011 and were neglected in this study. The original historical landcover map contained five land cover types (the urban area, cultivated land, forest, water, and desert). We classified the historical landcover map by only two types (cultivated land and other). The historical landcover map was then transformed from the original polygon to the raster format with the same spatial resolution of the Landsat data. For convenience, we added the ground truth data (the historical landcover map) to the Landsat dataset as the seventh band. After that, the TM images and corresponding ground truth were split into 256 × 256-pixel tiles to keep the memory consumption low during the training and validation. These tiles were adjacent and non-overlapping.

To evaluate different combinations of spectral bands on the performance of cultivated land extraction, we defined three datasets, namely, TM-NRG, TM-RGB, TM-All, with a varying number of spectral bands. An overview of each dataset is provided in Table 3. To avoid overfitting during training, we selected 4050 tiles (approximately 70%) randomly for training, 867 tiles (approximately 15%) as validation data for adjusting the model hyperparameters during training, and the remaining 868 tiles (approximately 15%) for independent testing. The methods we used for comparation (RF, U-Net, and U-Net++) were all based and tested on the same datasets.

## 4. Methodology

**Figure 2:**
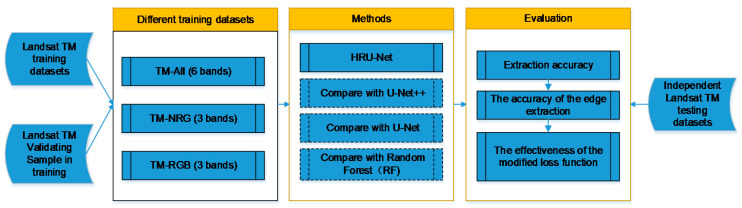
Overview of the performance evaluation framework. High-resolution U-Net (HRU-Net).

In this paper, a new end-to-end cultivated land extraction algorithm, high-resolution U-Net (HRU-Net), was proposed, with the aim to extract the same land-cover type with different spectra accurately and preserve the image details by improving the skip connection structure and loss function of the original U-Net. Figure 2 shows an overview of the workflow of this study.

### 4.1. The Original U-Net and U-Net++

**Figure 3:**
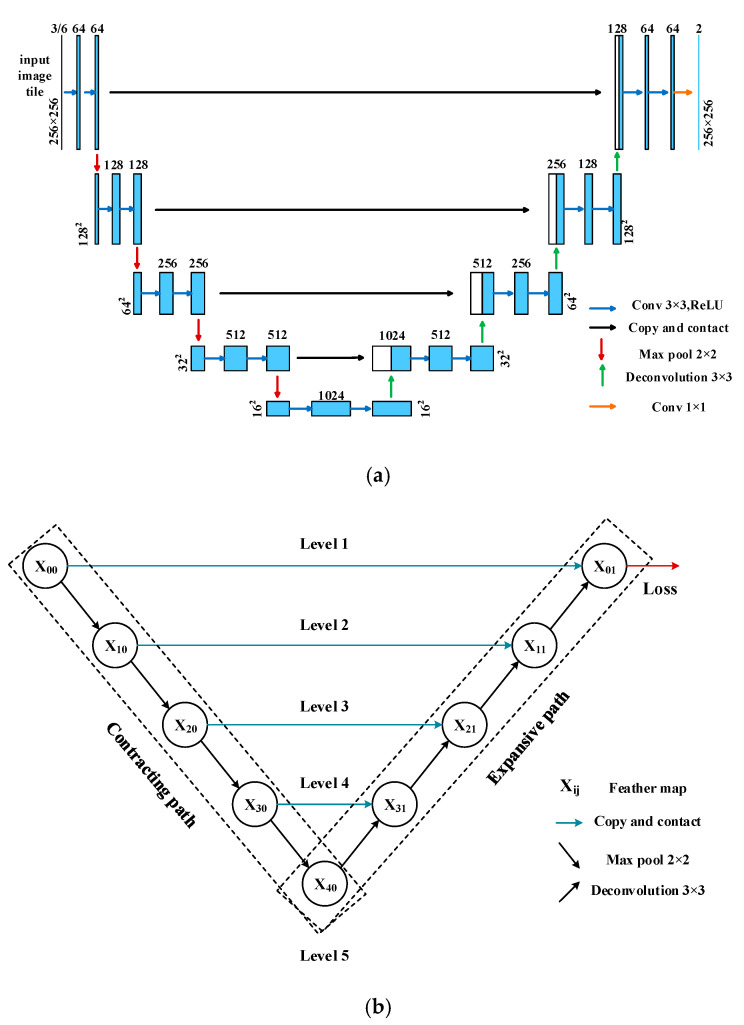
(**a**) U-Net architecture [28] and (**b**) Simplified U-Net topology diagram from (**a**).

Initially, the U-Net was developed for biomedical image segmentation. We chose it as the base network to extract cultivated land as it achieves state-of-the-art performance on benchmark datasets even with limited training data [27,28]. Figure 3a shows the structure of the original U-Net network. It contains two main pathways: the contracting pathway on the left side and the expansive pathway on the right side.

In the contracting path, the input image was first sent to the feature detection by operating a 2-dimensional convolution by the typical architecture of a convolutional network, which repeated the block of two 3×3 convolutions, a rectified linear unit, and a 2×2 max-pooling operation, iteratively. To enlarge the “sense field” of the convolution kernel and give the network more ability for a global view of the features of the object, the “pooling operation” was added to contract the feature map into the lower level. Meanwhile, a skip connection structure attempted to reduce the loss of image details in the “pooling operation” in the contraction path by adding a feature vector to the expansive path at the same level, as indicated by the gray arrow in Figure 3a.

In the expansive path, the central idea was to combine the low-level feature maps to expand the image size. First, the low-level feature map was up-sampled by a 2×2 transpose convolution. Secondly, the output was combined with the corresponding feature map from the skip connection at the same level. Thirdly, two 3×3 convolutions and the rectified linear unit (ReLU) activation function were applied for further feature detection.

At the final layer, to match the number of channels to the number of classes in the final output, a 1×1 convolution with the Softmax activation function was used. The output of this network was the predicted probabilities of each class p(x). The final class labels were calculated by selecting the highest probability class in the vector p(x). In this structure, the skip connection was the only path to restore the high-resolution details in every convolution level.

As shown in Figure 3b, in order to emphasize the skip connections between the feature maps at the different levels, the structure of the U-Net (Figure 3a) was simplified by replacing the convolution process in Figure 3a with the symbol $X_{ij}$, where i is the level index and j is the convolution node index at the same level. For example, the $X_{10}$ represented the first convolution module at the second level.

The other benefit of the U-Net is that the number of the trainable parameters is relatively small. Other networks, such as FCN and DeconvNet, are more complicated with more trainable parameters, and require a bigger training set and a longer time to train [35,37]. Usually, to reduce the training time of networks, a pre-trained network can be used to retrain the top layer on a new dataset. However, the pre-trained network is usually trained on natural pictures with RGB bands. As we hope to take full advantage of the multi-band data of remote-sensing images instead of only RGB channels, this strategy cannot work well when the channel difference happens between the pre-trained and the new datasets. For this reason, the U-Net network in this study was trained from scratch.

Under the hypothesis that the feature maps from contracting path (encoder networks) can enrich the prior for the expansive path (decoder networks), UNet++ was proposed to increase the segmentation accuracy for medical images [54]. In UNet++, a small down-triangle structure was designed as the basic unit. With this unit, UNet++ can be easily extended to different levels depending on the accuracy and performance required for the different tasks. The intuitive purpose of the UNet++ is to reach the high overall accuracy of segmentation in medical images for improving disease diagnosis. In this paper, we focused on the application of a deep learning model for satellite images, specifically to recover the edge details of the land cover types which were lost during the “pooling” process. More details of the HRU-Net will be described in the next section.

### 4.2. The High-Resolution U-Net

Giving the network the ability to learn the high-resolution details of the image is the key to solving the problems of insufficient accuracy of cultivated land extraction due to a loss of image details. The idea of the U-Net network is to learn and recover high-resolution details directly from a low-resolution feature map by simply combining the feature maps from the skip connection at the same level. In the first step, learning and recovering high-resolution information from the lower level feature map is extremely difficult as it requires the recovery of non-existent details. In the second step, simply adding the feature map from the skip connection to a low-level feature map could disturb the concise features learned from the low level. The image details from the skip connection are limited as it has already suffered the “pooling process” in the previous feature detection.

**Figure 4:**
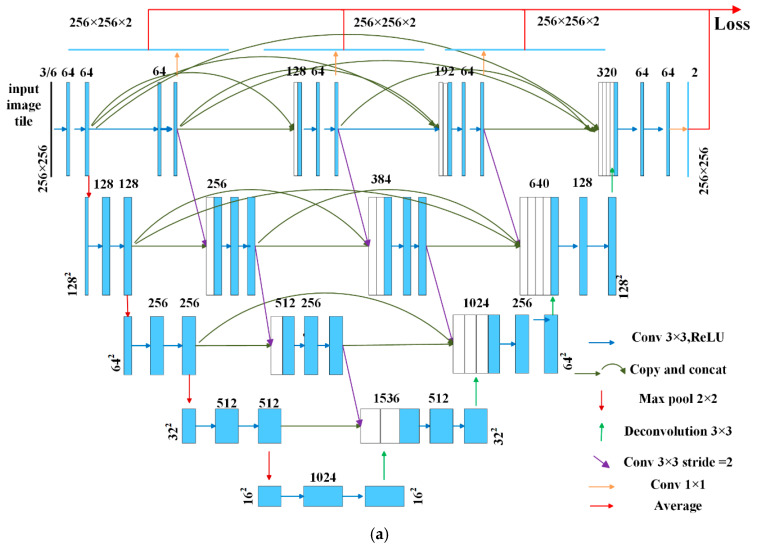

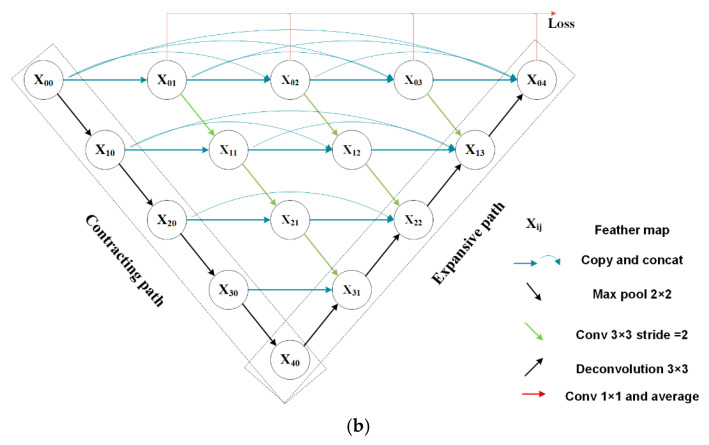
(**a**) The HRU-Net architecture and (**b**) the simplified topology diagram of the HRU-Net.

Considering the multi-level structure of the U-Net and the higher level that the convolutional nodes locate, a smaller number of the “pooling process” were applied to these nodes. As a result, more texture details remained in these feature maps. The key to solving this problem was to find a proper strategy to enrich the feature map details by involving information from the higher level and reducing the noise amplifying effect at the same time. The new structure we proposed in this study, the HRU-Net, used the idea of maintaining high-resolution details during the whole process to ensure that the multi-resolution descriptions of the image were always present (Figure 4).

In this structure, the image details not only came from the same level but were also enriched from the higher level. To reduce the noise from the higher level and produce more deep sematic features, several convolutional nodes were added in the skip connection path. The new convolutional nodes increased the number of overall parameters, so in this study, to learn the network parameters more efficiently, the idea of deep supervision was adopted to re-design the loss function. The network architecture is illustrated in Figure 4a. Compared to the original U-Net architecture, the HRU-Net kept the same structure in the contracting and expansive path. More skip connections were added between the contracting and expansive path. The simplified topology diagram of the HRU-Net is shown in Figure 4b, simplified from Figure 4a by replacing the convolution process with the symbol $X_{ij}$ to make clearer the structure of the skip connection in the HRU-Net.

In the following part, we will further discuss from the two perspectives: (1) how to improve the skip connection structure and (2) how to use the idea of deep supervision to design the loss function.

#### 4.2.1. Improving the Skip Connection Structure

The skip connections were first introduced in the FCN [37]. Starting from the FCN, this structure has been widely introduced in many models to retain the high-resolution details across the different levels. In the U-Net, the feature maps in the contracting path are directly sent to the expansive path by skip connections. To simply copy the feature map from the contracting path and merge to the expansive path with the feature map from the lower level does not always work as the details have already been lost before the skip connections. The basic idea to solve this problem is to borrow the image details from a higher level to minimize the effect of the “pooling” (the green-sampling arrow in Figure 4b). Followed by this idea, in the HRU-Net the skip connection was improved in the following two aspects:

(1) Maintained resolution details at the same level

First, the HRU-Net maintained feature maps at the same layer by applying a repeated convolution module (shown in blue arrows in Figure 4b). Each module consisted of two 3×3 convolutions and a rectified linear unit. Then, it incorporated shallow features into deep features at each layer by a skip connection at the same level to retain details (shown in blue curved arrows in Figure 4).

(2) Fused multi-scale details cross different levels

The HRU-Net converted the high-resolution feature map into the same size and the same number of channels as the lower-level required by applying a 3×3 convolution with a stride of 2 (shown in green arrows in Figure 4b); then, the HRU-Net combined this high-level feature map with the feature map from the previous node by a convolution operation and a concatenation operation; at last, two 3×3 convolutions and a rectified linear unit were applied for further feature detection (shown in blue arrows in Figure 4a,b).

The HRU-Net can be formulated as follows:

(1) $$X_{ij}=\left\{ \begin{array}{cc} c\left(d(X_{\left(i-1\right)j})\right) & j=0\\ c\left({\left[X_{ik}\right]}_{k=0}^{j-1}\right) & i=0,\;j=1,\;2,\;3\\ c\left(\left[{\left[X_{ik}\right]}_{k=0}^{j-1},u\left(X_{\left(i+1\right)\left(j-1\right)}\right)\right]\right) & i=0\;and\;j=4\\ c\left(\left[{\left[X_{ik}\right]}_{k=0}^{j-1},d\left(X_{\left(i-1\right)j}\right)\right]\right) & j>0,i>0\;and\;i+j<4\\ c\left(\left[{\left[X_{ik}\right]}_{k=0}^{j-1},d\left(X_{\left(i-1\right)j}\right),u\left(X_{\left(i+1\right)\left(j-1\right)}\right)\right]\right) & j>0,i>0\;and\;i+j=4 \end{array}\right.$$

where $X_{ij}$ is the output feature map of the node (i,j), where i is the level index and j is the convolution node index at the same level. Function c(·) represents the convolution operation, u(·) denotes an up-sampling operation, d(·) is a pooling or down-sampling operation, and [·] is the concatenation operation. The overall structure can be described as follows:

- The nodes at level j=0, $X_{i0}$ can be gained by only one input $X_{\left(i-1\right)0}$, which is from the previous layer in the contracting path. The max pooling and convolution operation are applied in nodes $X_{\left(i-1\right)0}$.
- The nodes at level i=0 and j<4 receive the j feature maps of the previous nodes at the same level. For example, $X_{03}$ can be gained by $X_{00}$, $X_{01}$, and $X_{02}$. The inputs are concatenated by concatenation operation, then the convolution operation is performed.
- The nodes at level i=0 and j=4 receive the j feature maps from the previous nodes at the same level and the up-sampled feature maps from the lower level. In particular, $X_{04}$ can be gained by $X_{00}$, $X_{01}$, $X_{02}$, $X_{03}$ and $X_{13}$. The up-sampled $X_{13}$ is concatenated with $X_{00}$, $X_{01}$, $X_{02}$, $X_{03}$ nodes.
- The nodes at the middle of the network, where j>0,i>0 and i+j<4, receive j+1 inputs (j inputs from are the j feature maps form previous nodes at the same level, one input is the down-sampled output from the higher level).
- The nodes at the end of each layer, where j>0,i>0 and i+j=4, receive j+2 inputs, (j inputs are from the j feature maps form previous nodes at the same level, one input is the down-sampled output from the higher-level, and one input is up-sampled output from the lower-level).

#### 4.2.2. Using the Idea of Deep Supervision to Modify the Loss Function

When designing the input of the loss function, the U-Net only obtains the classification probabilities from $X_{04}$. Compared to the U-Net, the HRU-Net generated full-resolution feature maps from multiple levels, $\left\{X_{0j},\;j\in \left(1,2,3,4\right)\right\}$, which can be used to apply deep supervision. We first obtained the classification probabilities at different semantic levels, from $\left\{X_{0j},\;j\in \left(1,2,3,4\right)\right\}$, through 1×1 convolutions with the Softmax activation function (as marked by red arrows in Figure 4), and then obtained the predicted class probabilities P(x) by averaging all probabilities,

(2) $$P\left(x\right)={\left[P_{0}\left(x\right),P_{1}\left(x\right)\right]}^{T}$$

where $P_{i}\left(x\right)$ is the predicted probability of x belonging to class i (i=0 for cultivated land, and i=1 for non-cultivated land). The class label y of a given image can be calculated by obtaining the label from the maximized probability in P(x):

(3) y=argmax(P(x)).

The loss function of HRU-net is defined as

(4) $$H\left(Y,\bar{Y}\right)=-\frac{1}{N}\sum_{i}Y_{i}log\left({\bar{Y}}_{i}\right)$$

with ${\bar{Y}}_{i}$ and $Y_{i}$ denoting the predicted and the actual probability of class i, respectively, and N being the batch size.

#### 4.2.3. Assessment

The accuracy evaluation metrics in this paper include (1) the overall accuracy, (2) Cohen’s kappa coefficient, and (3) the F1-score. The overall accuracy is defined as the number of correctly classified pixels over the total number of pixels. It is simple and intuitive but may fail to assess the performance thoroughly when the number of samples for different classes varies significantly. Cohen’s kappa coefficient is more robust, as it takes into consideration the possibility of agreements occurring randomly. Let $p_{0}$ be the percentage of pixels correctly classified, and $p_{e}$ be the expected probability of agreement when the classifier assigns class labels by chance, Cohen’s kappa coefficient is defined as:

(5) $$K=\frac{p_{0}-p_{e}}{1-p_{e}}.$$

Usually, we characterize K<0 as no agreement, [0,0.20] as poor agreement, [0.20,0.40] as fair agreement, [0.40,0.60] as moderate agreement, [0.60,0.80] as good agreement, and [0.80,1] as almost perfect agreement. The F1-score is defined as the harmonic mean of the precision rate and recall rate:

(6) $$F1=\frac{2\times P\times R}{P+R}$$

where P is the number of positive classes predicted correctly (*TP*) divided by the number of all positive results (including both true positive *TP* and false positive *FP*), and R is the number of true positive results (*TP*) divided by the number of all relevant samples (true positive plus false negative *FN*):

(7) $$P=\frac{TP}{TP+FP}$$

(8) $$R=\frac{TP}{TP+FN}$$

An F1 score reaches its best value at 1 (perfect precision and recall) and its worst at 0.

## 5. Results and Discussion

### 5.1. The Learning Process of the HRU-Net

In this study, we hoped to make full use of the advantage of the multi-band data of remote-sensing images instead of only RGB images. Thus, we decided to train the all network (HRU-Net, U-Net++, the original U-Net, and RF) from scratch. To compare the performance of the different numbers of bands, three datasets were prepared (Table 2). The performance of the near-infrared (NIR) band can be analyzed when comparing the results of the TM-NRG with those of the TM-RGB. Similarly, comparing the results from the TM-All to the TM-NRG datasets, the improvement of the shortwave-infrared (SWIR) can be investigated.

The HRU-Net, U-Net++, U-Net, and RF were trained and tested on the three datasets (Table 2) separately. In each dataset, all samples were randomly split into three: the training set, the validation set, and the testing set. The training set was used for model training. The validation set was used to calibrate the hyperparameters of the deep learning model, and the testing set was used to apply the independent assessment for the different models.

**Figure 5:**
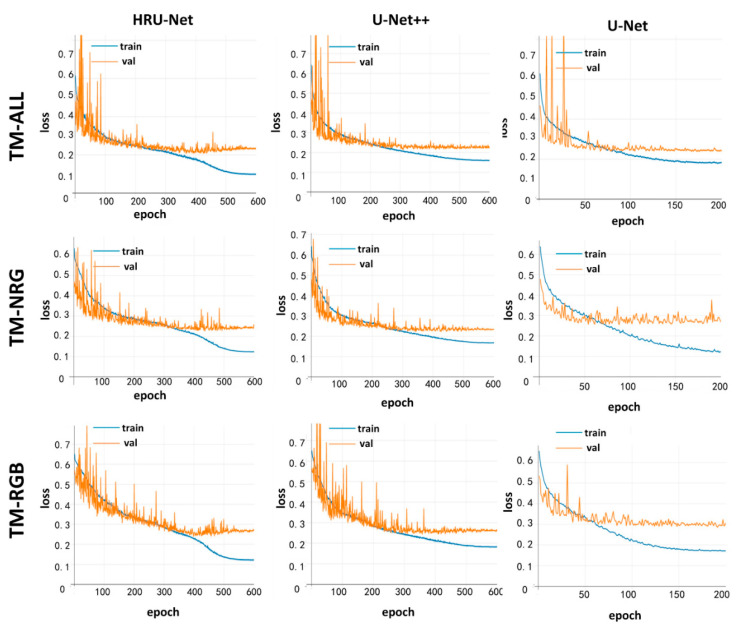
Visualizations of the training history for the HRU-Net, U-Net, and U-Net++ models.

All experiments of the HRU-Net, the U-Net++, and U-Net were carried out on four TITAN X GPUs. We used PyTorch backend as the deep-learning framework (https://pytorch.org/). To maximize the GPU memory usage, we set a different batch size for each network (HRU-Net and U-Net++:24, U-Net:48), and each network model was trained by starting with a different initial learning rate (HRU-Net:0.0015, U-Net++:0.002, U-Net:0.0002). For three networks, the gradient descent optimization (SGD) optimizer with a momentum of 0.95 and a weight decay of $10^{-4}$ was adopted, and the learning rate was decreased every iteration by a factor of $0.5\times \left(1+cos\left(\pi \frac{iter}{max\;iters}\right)\right)$. The batch-norm parameters were learned with a decay rate of 0.9, and the input crop size for each training image was set to 256×256. Figure 5 shows the training history of the HRU-Net, U-Net++, and U-Net. Considering the popularity and the success of the RF in the classification of remote-sensing images, we also trained the traditional RF classifier on the same datasets as a comparison. The Scikit-learn (http://scikit-learn.org, 2018) implementation was adopted for RF in our experiments, which employed several optimized *C4.5* decision trees to improve the prediction accuracy while controlling the over-fitting at the same time [55]. The detailed parameters of the random forest are shown in Table 4.

The visualizations of the training history for the HRU-Net, U-Net, and U-Net++ models are shown in Figure 5. The blue line represents the loss calculated by the training set at each epoch. The orange line represents the loss calculated by the validation set at each epoch. Both values of the loss are high at the beginning of the training process. As the model developed by each epoch, both loss values decrease. The main purpose of Figure 5 is to avoid overfitting during the training. As shown in Figure 5, all orange lines converge to a certain value, indicating that there is no overfitting that happens during the training process. In other words, all three models were sufficiently trained and can be compared fairly with each other.

### 5.2. Comparation of the HRU-Net with U-Net, U-Net++, and RF

We tested the results of the HRU-Net, U-Net, U-Net++, and RF from three aspects: (1) the overall accuracy, (2) the accuracy of the edge details, and (3) the robustness for the intra-class variation.

#### 5.2.1. The Overall Extraction Accuracy

**Figure 6:**
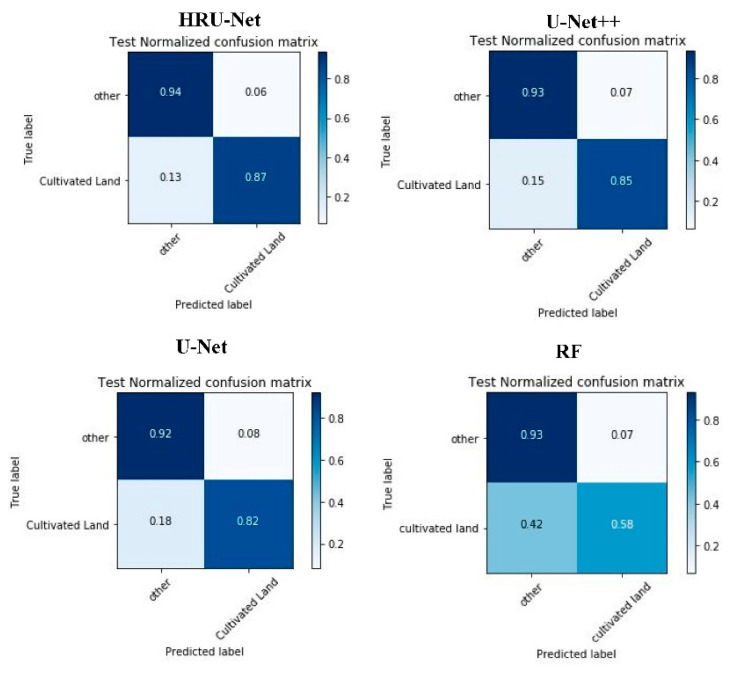
Confusion matrix for the HRU-Net, U-Net++, U-Net, and RF models over the independent test dataset for the Landsat TM-All dataset.

Table 5 and Figure 6 show the assessment of each method on the independent testing datasets. Over the three datasets, the HRU-Net outperformed the other three models concerning the overall accuracy (Acc), Cohen’s kappa coefficient (K), and F1 score (F1).

First, the results in Table 5 indicate that the NIR and SWIR bands could significantly improve the overall accuracy by 1–4%. The TM-All dataset achieved the highest accuracy compared to the results from the TM-NRG and TM-RGB datasets. The highest improvement appeared when adding NIR to the RF model (3.35%). This may be related to the model capacity to capture the higher-scale features (such as the possible nonlinear band combinations). As the deep learning model can do better at this perspective, less improvement appears when adding the new bands for training.

Secondly, the HRU-Net achieved the highest extraction accuracy in all three datasets. Especially on the TM-All dataset, the HRU-Net achieved an overall accuracy of 92.81%, improved by 1.07% compared with U-Net++, 2.98% to U-Net, and more than 16% compared with RF. The HRU-Net had the best kappa coefficient of 0.75–0.81, increased by 0.01–0.02 compared with U-Net++, 0.07–0.09 compared with U-Net and 0.33–0.50 compared with RF. A similar result can be found in the F1 score.

Thirdly, as we can see from the Table 5, the NIR band and the SWIR band can provide some useful features to help to distinguish the cultivated land and others, but the improvement was bigger in the RF model (1–4% improvement in Acc) rather than in deep learning models (0.4–1% improvement in Acc). One possible reason could be that the deep learning models have more learning capacity which can extract deeper level features such as the shape and gradients. The other reason could be that under the high intra-class spectral variation, the benefit of the NIR and SWIR band to separate the vegetation and non-vegetation pixels is less effective to distinguish the cultivated land and non-cultivated land since cultivated land can be covered by vegetation or not during the different times.

Figure 6 shows the confusion matrix for the three models over the TM-All dataset. The results indicated the HRU-Net achieved the highest recall and precision. The type 1 and type 2 error in the HRU-Net also remained the lowest compared to the U-Net++, U-Net, and RF.

Table 6 shows the overall accuracy of the HRU-Net under 50%, 60%, and 70% training sets. As we expected, the smaller training set, the lower the accuracy will be, but as we can see, even with the 50% training samples, the accuracy decreases slowly in HRU-Net.

Table 7 shows the time consumption during the training of the HRU-Net, U-Net++, and U-Net. The RF is excluded as it was trained by CPU rather than the GPU; thus, it is not comparable to the other three GPU-based algorithms. Compare to the original U-Net, the training time increased approximately 2.6 times as more model parameters were involved by adding more complex skip connections. The time consumption of the HRU-Net was similar to the U-Net++ as these two networks had a similar number of parameters when the level was the same.

#### 5.2.2. The Accuracy of the Edge Details

**Figure 7:**
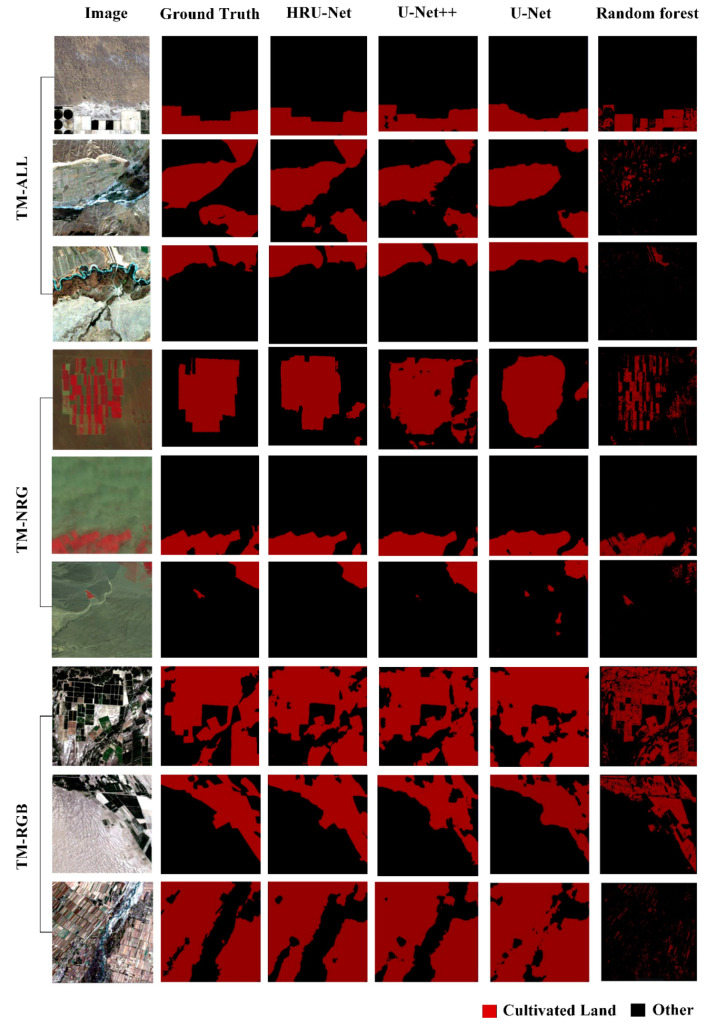
Selected results for the best RFU-Net, U-Net, and random forest models on all three datasets.

As shown in Figure 7, the accuracy of the edge details was evaluated by visual interpretation. The results of the HRU-Net had clearer edges and richer details than those of the U-Net++ and U-Net. Specifically, comparing with the U-Net++, the more detailed edge remained in the output. The edge of the output from the HRU-Net was much more accurate than the edge of the original U-Net, as the loss of details could not be recovered from the lower nodes in the U-Net. In the output of the RF, the edge was sharp. However, the farmland without the crop covering was not detected correctly as it suffered from intra-class variation.

**Figure 8:**
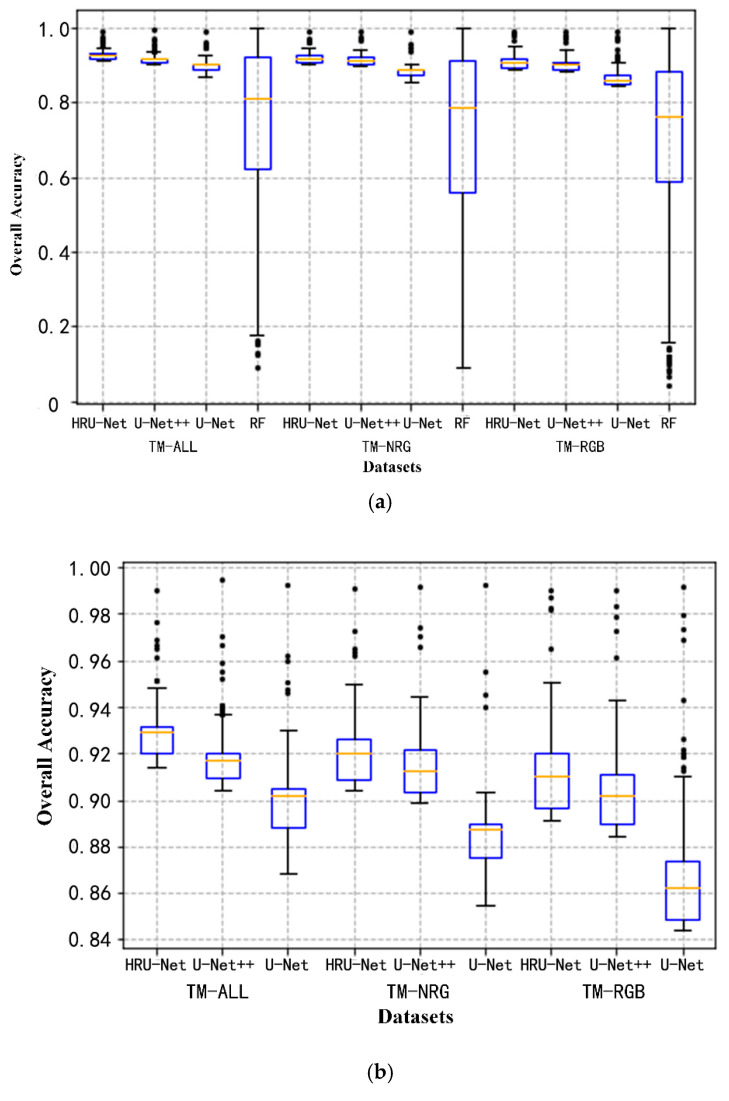
Boxplot of the overall accuracy distribution over the test dataset (868 tiles). (**a**) The comparison between RF and deep learning algorithms. (**b**) The comparison among HRU-Net, U-Net++, and U-Net.

The robustness for the intra-class variation for the different models can be seen in Figure 8. In Figure 8, the overall accuracy of each tile in the testing dataset was plotted. The different tiles were randomly located and captured the main spectral variation of the cultivated land. The variation of the overall accuracy can be seen as the performance of the model handling the intra-class variation. As shown in Figure 8a, the RF model had the highest variation as indicated by its limited generalization ability to cross different spectra. Figure 8b shows a clearer comparison among the HRU-Net, U-Net++, and U-Net by removing RF from Figure 8a. In Figure 8b the variation of HRU-Net is similar to the U-Net++, however, it achieves higher accuracy in all three datasets. This indicates the effectiveness of the HRU-Net for solving the intra-class variation problem for accurate classification.

#### 5.2.3. The Effectiveness of the Modified Loss Function in the HRU-Net

**Figure 9:**
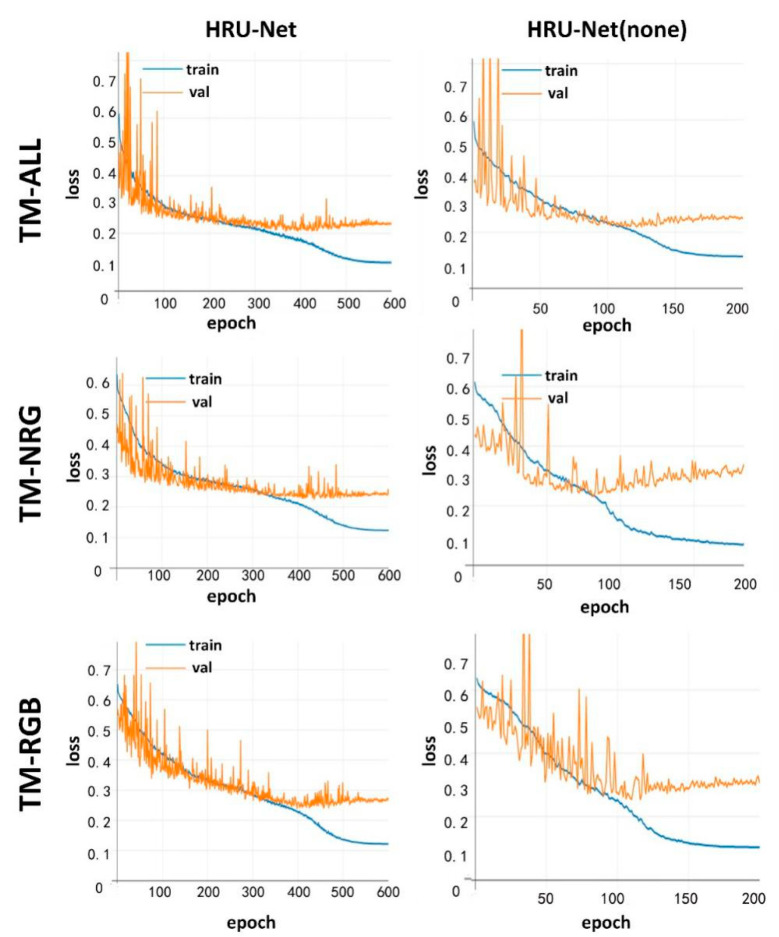
Visualization of the training history for the HRU-Net. HRU-Net (none) represents the HRU-Net without the modification of the loss function.

To clarify the effectiveness of the modified loss function, we compared the HRU-Net with the modified loss function and the HRU-Net with the original loss function designed by U-Net. Figure 9 shows the difference in the training history of these two models. The HRU-Net with the modified loss function can be trained with more epochs; the slight overfitting happened after 500 epochs compared to after 125 epochs with the original U-Net loss function. In all three datasets, the HRU-Net with the original U-Net loss function (right column) appeared to have quicker overfitting, which was expected when training with a smaller number of bands (such as the TM-NRG and TM-RGB datasets).

Table 8 shows the overall accuracy compared with or without the modified loss function. The results indicated that the modified loss function contributed nearly 4–5%, 5–16%, and 2–8% improvement of the overall accuracy, kappa, and F1 score over the three datasets. This indicates the modified loss function in the HRU-Net can help the model learn the spectral features of cultivated land more effectively from any perspective.

### 5.3. Discussion

How to fix the smooth effectiveness of the pooling process to maintain or recover the image details for the deep learning model has become a topic of concern in recent years. The model we presented in this study followed the idea of maintaining and enhancing the image details during all convolution processes. The structure of the HRU-Net was similar to the 5-level U-Net++; however, the initial purposes were different. As mentioned before, the HRU-Net aimed to maintain and transfer the image details from shallow to deep levels. However, the purpose of the U-Net++ was to balance the speed and accuracy by redefining the original U-Net structure with the combination of the basic down-triangle units to achieve a more flexible structure for the different sizes of the network. The difference is that in U-Net++ more feature maps from lower levels were merged rather than higher-levels feature maps being combined in HRU-Net.

At a 30 m scale, the spectral mixing pixel is one of the sources of the classification uncertainty. The model, such as endmember extraction or mixed pixel decomposition, could help this situation. Fortunately, for the study area of this study, Xinjiang China, cultivated land is located in the huge flat area near the river or lake. The farmland is adjacent rather than separated, so the influence of the mixing pixels relatively low. This problem could be more serious when applying this model in more broken farmland, such as the southeast province of China.

**Figure 10:**
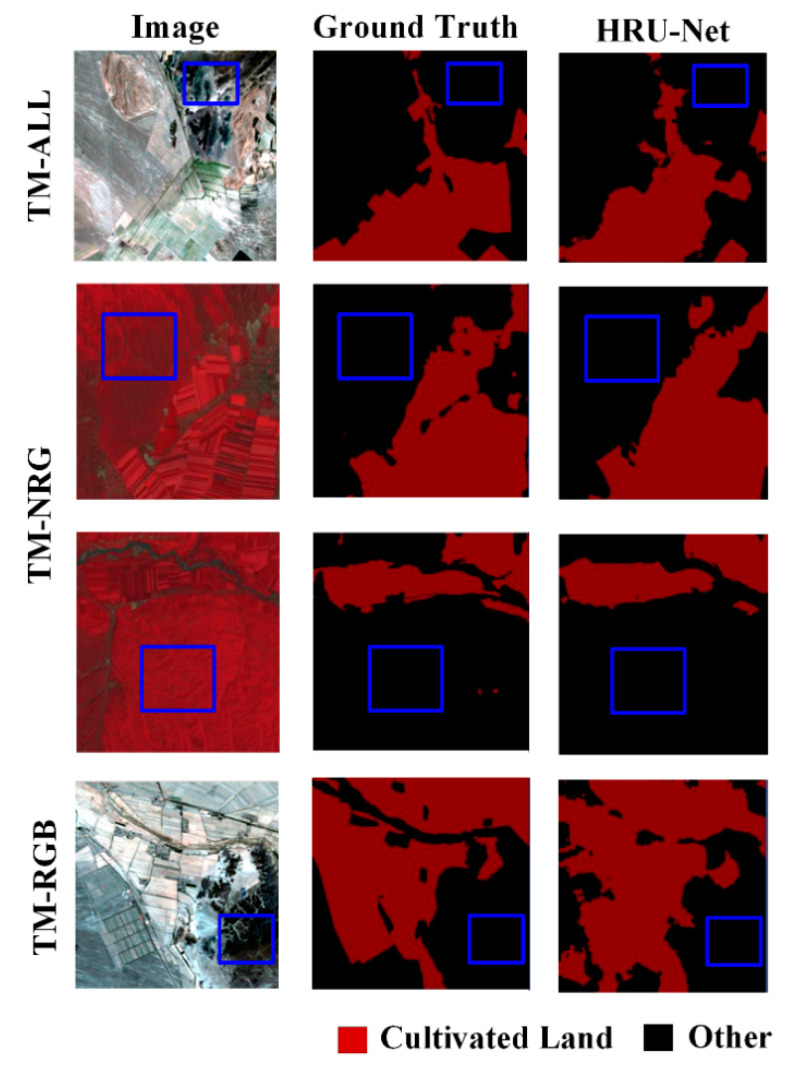
Visual investigation the classification accuracy of the HRU-Net under the vegetated area.

The experiment in this study basically is a binary decision which mainly classify the cultivated land versus other (everything else). One of the questions is whether all the other vegetated areas (but non-cultivated) like grass fields or forest plots are well separated and classified as non-cultivated. To answer this question, we further evaluate the classification accuracy of the HRU-Net under the vegetated area. As we can see from Figure 10, all vegetated areas (grassland and forest) are correctly classed to the “others” category. This indicated the deep features from the spectral, texture, and time series may help the deep learning model like HRU-Net to better distinguish the cultivated land with other vegetated land cover.

In this study, we used three years of data to capture the spectral variation of the cultivated land under different conditions. The labels of the training and testing data were obtained from historical landcover maps and manual interpretation of the corresponding satellite images. They may contain errors as the accuracy depended on the performance of a human analyst. In particular, regarding the accuracy of the edge and cultivated land extraction with different spectra, interpretation and delineation of cultivated land could be partially subjective.

More accurate extraction could be achieved by involving more prior knowledge, such as the time-series features of the cultivated land or by enhancing the spectral features of the soil or crops by adding the vegetation index as auxiliary channels.

## 6. Conclusions

In this study, we proposed a new end-to-end cultivated land extraction algorithm, the high-resolution U-Net (HRU-Net). Compared with the original U-Net, the HRU-Net had two improvements: (1) it improved the skip connection structure, and (2) it used the idea of deep supervision to modify the loss function. We tested the proposed method and compared it with the U-Net++, U-Net, and the RF on three Landsat TM datasets with different spectral band combinations and drew the following conclusions:

(1) The NIR and SWIR band improved the extraction accuracy of the cultivated land extraction. This follows the commonsense idea that more independent features can better help with class separation.
(2) Due to the high intra-class variation of the cultivated land, the traditional machine learning RF model had a high variation in the classification accuracy. This may be related to the Hughes phenomenon when more divergent features are involved in the model.
(3) The edge details were improved by the new structure of the HRU-Net. The HRU-Net model achieved the best results in all three Landsat TM images datasets with the lowest accuracy variation for the difference spectra of the cultivated land.

The HRU-Net model presented in this study demonstrated good performance in extracting the target with high edge details and high intra-class spectral variation. This model can be further used to extract the target within these characteristics. The model introduced in this study can be extended or combined to more other high spatial resolution satellite data, such as Sentinel-2, GF1, and GF2.

## Figures and Tables

**Table 1:** Parameters of Landsat4—5 thematic mapper (TM).

Sensor	Bands	Wavelength/μm	Resolution/m
Landsat4—5 TM	B1-Blue	0.45–0.52	30
	B2-Green	0.52–0.60	30
	B3-Red	0.63–0.69	30
	B4-NIR	0.76–0.90	30
	B5-SWIR1	1.55–1.75	30
	B6-TIR	10.40–12.5	120
	B7-SWIR2	2.08–2.35	30

**Table 2:** Data list of Landsat 5 top of atmosphere (TOA) products used in this study.

Date	Area	Image File Names	Path/Row
10 Jun 2010	Bosten farmland	LT51420312010161IKR00.tar	142/31
13 Aug 2010		LT51420312010225IKR00.tar	
2 Sep 2010		LT51430312010248IKR00.tar	143/31
26 Aug 2009		LT51420322009238IKR00.tar	142/32
27 Sep 2009		LT51420322009270KHC00.tar	
15 Jul 2011		LT51420322011196IKR00.tar	
3 Oct 2011		LT51420322011276KHC01.tar	
29 Aug 2010		LT51420322010241IKR00.tar	
30 Sep 2010		LT51420322010273IKR00.tar	
20 Aug 2010		LT51430322010232IKR00.tar	143/32
4 Aug 2010		LT51430322010216IKR00.tar	
21 Sep 2010		LT51430322010264IKR00.tar	
11 Aug 2010		LT51440312010223IKR01.tar	144/31
15 Nov 2010		LT51440312010319KHC00.tar	
11 Aug 2010	Urumqi farmland	LT51440292010223IKR01.tar	144/29
15 Nov 2010		LT51440292010319KHC00.tar	
11 Aug 2010		LT51430292010232IKR00.tar	143/29
21 Sep 2010		LT51430292010264IKR00.tar	
4 Jun 2011		LT51430292011155IKR00.tar	
7 Aug 2011		LT51430292011219KHC01.tar	
23 Aug 2011		LT51430292011235KHC01.tar	

**Table 3:** Three different TM datasets used in this study.

Dataset	Bands	Resolution/m	Training Sample	Validating Sample in Training	Testing Sample
TM-NRG	NIR, Red, Green	30	4050 (70%)		868 (15%)
TM-RGB	Red, Green, Blue	30		867 (15%)	
TM-All	Blue, Green, Red, NIR, SWIR1, SWIR2	30			

**Table 4:** The parameters used in the random forest algorithm.

Parameter	Description	Value
n_estimators	Max number of the decision trees	160
criterion	The principle function used to separate a branch	Gini
max_features	Max number of the features considering when separating a branch	All
max_depth	Max depth of the tree	No limit
min_samples split	The minimum number of samples at least remains in one node that can be split.	10
min_samples leaf	The minimum number of samples at least remains in leaf nodes.	1

**Table 5:** Extraction accuracy of HRU-Net, U-Net++, U-Net, and RF.

	TM-All Dataset			TM-NRG Dataset			TM-RGB Dataset		
	Acc.	K	F1	Acc.	K	F1	Acc.	K	F1
HRU-Net	**92.81**	**0.81**	**0.90**	**92.01**	**0.79**	**0.89**	**91.05**	**0.75**	**0.88**
U-Net++	91.74	0.79	0.89	91.31	0.78	0.88	90.31	0.74	0.86
U-Net	89.83	0.74	0.86	88.47	0.72	0.85	86.33	0.66	0.82
RF	76.13	0.48	0.69	75.22	0.36	0.66	71.87	0.25	0.61

**Table 6:** The overall accuracy of the HRU-Net under 50%, 60% and 70% training sets.

	Percentage of the Training Data	TM-All Dataset			TM-NRG Dataset			TM-RGB Dataset		
		Acc.	K	F1	Acc.	K	F1	Acc.	K	F1
HRU-Net	50%	89.16	0.75	0.88	88.65	0.74	0.87	86.62	0.70	0.85
	60%	89.70	0.76	0.88	88.99	0.73	0.87	86.68	0.67	0.84
	70%	92.81	0.81	0.90	92.01	0.79	0.89	91.05	0.75	0.88

**Table 7:** The time consumption and network complexity of the training of the HRU-Net, U-Net++, and U-Net.

	Network Complexity	TM-All Dataset		TM-NRG Dataset		TM-RGB Dataset	
	Number of Free Parameters	s/epoch	epoch	s/epoch	epoch	s/epoch	epoch
HRU-Net	3.85 × 10^7^	102.08	600	98.84	600	98.47	600
U-Net++	3.62 × 10^7^	107.44	600	103.09	600	103.47	600
U-Net	1.34 × 10^7^	39.23	200	36.79	200	36.92	200

**Table 8:** Comparison of the HRU-Net with or without the modified loss function.

	TM-All Dataset			TM-NRG Dataset			TM-RGB Dataset		
	Acc.	K	F1	Acc.	K	F1	Acc.	K	F1
HRU-Net with the new loss function	**92.81**	**0.81**	**0.90**	**92.01**	**0.79**	**0.89**	**91.05**	**0.75**	**0.88**
HRU-Net with original U-Net loss function	90.63	0.76	0.88	87.80	0.63	0.81	87.30	0.68	0.84
